# Battling Unawareness of One’s Test Performance: Do Practice, Self-Efficacy, and Emotional Intelligence Matter?

**DOI:** 10.3390/bs13030275

**Published:** 2023-03-21

**Authors:** Maura A. E. Pilotti, Khadija El Alaoui, Arifi N. Waked

**Affiliations:** College of Sciences and Human Studies, Prince Mohammad Bin Fahd University, P.O. Box 1664, Al Khobar 31952, Saudi Arabia

**Keywords:** performance estimation, confidence, metacognition, Middle Eastern students

## Abstract

The “Dunning–Kruger effect” refers to the tendency of poor performers to overestimate test outcomes. Although a widespread phenomenon, questions exist regarding its source and sensitivity to countermeasures. The present field study aimed to (a) examine whether practice with tests used in previous classes can enhance students’ ability to estimate test outcomes, (b) determine the main source of the effect (i.e., is it unawareness of one’s readiness or wishful thinking?), and (c) assess the extent to which particular individual differences can be used as predictors of test performance. In this study, participants practiced with old tests and then completed the final exam. Before and after the exam, they predicted their grades and indicated their subjective confidence in the predictions made. Furthermore, participants’ emotional intelligence and self-efficacy about their academic abilities were surveyed. Results suggested that poor performers were not unaware of their test preparation, but rather engaged in wishful thinking. In fact, although they overestimated their test grades, their estimates not only improved after completing the final test but also were regarded with little confidence. Overall, estimation bias was a good predictor of students’ final test performance, whereas subjective confidence and emotional intelligence only weakly predicted such performance. Thus, if proactive interventions are to be developed for at-risk students, performance-estimation tasks may offer valuable information regarding such students’ future performance in a course much more than emotional intelligence and self-efficacy measures.

## 1. Introduction

Some time ago, several articles emerged in the extant literature regarding the so-called “Dunning–Kruger effect” [1]. In educational settings, the effect refers to students’ estimates of their academic performance. For instance, if students are asked to predict their grades on a test, poor performers will tend to overestimate how well they will perform, whereas higher performers will yield largely unbiased estimates or just minor biases toward underestimation [1,2,3,4,5]. The effect has been attributed to poor performers who exhibit little insight into their shortcomings, presumably because their difficulties are twofold. Deficiencies in their knowledge prevent them from both performing well (a behavioral outcome) and recognizing that their answers to tests and assignments are less than adequate (a metacognitive outcome). The effect has been questioned on two main fronts. First, it has been proposed that the phenomenon may be the byproduct of statistical restrictions in the range of available grades [6]. That is, poor performers, who find themselves at the lower-end tail of the grade distribution, would be faced with a floor effect fostering overestimation. Instead, higher performers, who find themselves at the upper-end tail of the grade distribution, would be faced with a ceiling effect fostering underestimation (for a rebuttal see [7]). Second, recent evidence has questioned the conventional interpretation of the effect. For instance, although students from different parts of the world tend to conform to the pattern of estimations that defines the Dunning–Kruger effect, poor performers may also be less confident in their test predictions than higher performers [8,9,10,11]. Poor performers may also exhibit less self-efficacy (i.e., overall confidence in their abilities) than students performing well [11]. Taken together, these findings have been interpreted as suggesting that poor performers face a serious dilemma when thinking about their performance on a test. They are aware of their deficiencies but do not possess sufficient confidence in themselves to address such deficiencies. Thus, they resort to wishful thinking (otherwise known as desirability bias) to find temporary comfort in an unlikely positive outcome. Wishful thinking is merely the desire for a particular outcome, which drives people to inflate their hope for that outcome [12].

In the studies by Hamann, Pilotti, and Wilson [9] and Pilotti, et al. [11], the interpretation of the Dunning–Kruger effect has been further put to the test by asking students to estimate their final test grades both before and after having completed the final test. The authors hypothesized that if indeed poor students were unaware of their performance, estimates and confidence in such estimates would not benefit from the reality check offered by the experience of completing the test. In agreement with this hypothesis, Pilotti et al. found that poor performers make estimates that are more accurate and less confident after the test. Post-test recalibration in estimation and confidence judgment suggested that the experience of taking a test can curb the wishful thinking of poor performers. It also depicted poor performers as searching for “a compromise between the wish to reach a particular conclusion and the plausibility of that conclusion given the available data” [13], (p. 569). Yet, Hamann, Pilotti, and Wilson [9] did not find post-test recalibration. Although a floor effect may have inoculated poor performers’ responses from the impact of test experience, other findings have questioned the pliability of the Dunning–Kruger effect. For instance, Miller and Geraci [14], reported that incentives alone (e.g., the opportunity to earn extra credit for accuracy in forecasting) fail to improve students’ estimations of their upcoming test performance, whereas explicit feedback (e.g., reminders of students’ individual test scores and predictions made) has a minor but noticeable beneficial effect. Instead, Osterhage [4] found that practice tests do not mitigate the Dunning–Kruger effect. Low performers continue to overestimate their test scores, whereas higher performers underestimate their test performance.

Notwithstanding the debate surrounding the pliability of the Dunning–Kruger effect, the extant literature underscores the broad influence of desire on human information processing. Desire can shape what one remembers of past grades [15], and more broadly, what one perceives [16], estimates [17,18], and judges [19]. Thus, the purported wishful thinking upon which the overestimation of poor performers is assumed to rest can have noticeable deleterious implications for test performance. Namely, students are unlikely to address their deficiencies and find remedies, thereby perpetuating a history of defeat.

Across the globe, educators and support staff are concerned about their ability not only to identify students at risk early enough to counteract otherwise-avoidable failures and withdrawals but also to implement effective remedies [20]. Thus, the sources of motivation and regulation of students’ behavior are invaluable information for educators and support staff. Among the various theories that attempt to explain the processes that drive and regulate behavior, the social–cognitive theory of Bandura [21] posits a combination of external and internal sources [22,23,24]. Among the latter, in addition to undesirable proclivities related to poor performance, dispositions exist that are believed to be capable of predicting students’ satisfactory performance. One such disposition is academic self-efficacy, broadly defined as students’ judgment of their capabilities to achieve the desired educational goals [25]. Self-efficacy beliefs are thought to lead students to suitable performance levels by enhancing their commitment, effort, and perseverance [26] and by reducing the occurrence of negative emotions, such as anxiety [27,28]. Not surprisingly, high levels of self-efficacy lead students to attribute undesirable outcomes to factors that can be controlled (e.g., effort), while low self-efficacy leads students to attribute such outcomes to their low abilities [29]. As a result of its motivating properties, academic self-efficacy has often been found to be positively correlated with academic performance [25]. However, null or inconsistent results also exist [25,30,31].

Another factor that is believed to be linked to students’ satisfactory performance is trait-emotional intelligence (also named emotional self-efficacy). Trait-emotional intelligence pertains to individuals who deal effectively with their emotions and those expressed by others [32]. Effectiveness includes the accurate perception and understanding of complex affective states of oneself and others, the successful regulation of affective expressions, and the capacity to use emotions to inform reasoning [33]. High trait-emotional intelligence characterizes individuals who understand their emotions and those of others and who can regulate emotions to foster well-being [32,34]. In academic settings, emotional intelligence, including greater emotional regulation and adaptability, is seen as helpful in coping with stress and thus in achieving academic success [35]. Broadly speaking, emotional intelligence has been assumed to impact academic success by helping learners navigate effectively the complexities of educational endeavors [36]. Not surprisingly, emotional intelligence has been linked to particular characteristics of learners that may directly or indirectly contribute to academic success. Among such features, one may find learners’ need for achievement [37], adaptive coping strategies [38], sense of psychological well-being [39], quality of interpersonal relationships [37,40,41], and conflict resolution competencies [42]. As for self-efficacy, emotional intelligence is often linked to satisfactory academic performance [43,44,45] but weak or null findings are not a rarity [46,47,48]. For instance, high levels of emotional intelligence may lead learners to be acutely aware of stressors and ensuing stress in their lives, which may contribute to higher perceived stress [49]. Deficient emotional regulation as well as cognitive interference and distraction may then ensue [50,51,52] with deleterious effects on performance [53].

## 2. The Present Study

This research was thought of as a field study to be conducted in the classroom with real students who were asked to estimate their actual grades on the final test (i.e., a discrete outcome prediction) and judge their subjective confidence in the estimations made (i.e., a confidence judgment). To counteract the purported Dunning–Kruger effect (i.e., lack of awareness of one’s readiness for a test as linked to poor performance on that test), prior to the final test, each student was given extensive training with tests used in previous semesters. Practice simulated the administration of a real test with detailed feedback provided after students individually answered each test question. Its content ensured that students would be familiar with the materials and the demands of the upcoming test. Feedback served as an external source of appraisal that was relevant to each student’s self-evaluation. When receiving specific feedback, students had the opportunity to assess their progress concerning goal attainment. Moreover, feedback offered students the opportunity to modify individual actions and strategies, thereby potentially shaping the selection of learning strategies and behaviors used to attain particular goals [54]. Under these conditions, we hypothesized that if unawareness was still the driving force of the purported Dunning–Kruger effect, the magnitude of the estimation bias and confidence in the estimations students made would both be inversely related to final test grades. That is, lower grades would be associated with increased overestimation and subjective confidence. Alternatively, if wishful thinking was the driving force of the effect, lower grades would be accompanied by increased overestimation and decreased confidence. That is, the magnitude of the estimation bias would be inversely related to students’ final grades, whereas confidence in the estimations made would be positively related to final test grades.

In addition to hypotheses about patterns of relationships between performance and outcome predictions or subjective confidence in such predictions, we examined two dispositions that, according to the extant literature, would be expected to be linked to desirable academic performance. To this end, we selected academic self-efficacy, as a global measure of students’ subjective confidence in their academic abilities, and emotional intelligence, as a global measure of the extent to which feedback from the practice session would be processed (i.e., a key feature of emotional intelligence; Refs. [55,56]). The magnitude of the estimation bias was expected to predict lower grades on the final test, whereas self-efficacy and emotional intelligence were expected to forecast higher grades. Whether the magnitude of students’ subjective confidence in the estimations made would forecast either higher or lower grades was thought to depend on whether students were driven by unawareness of their readiness for the test or wishful thinking. Nevertheless, the extent to which each of the selected factors would independently contribute to performance was a matter to be discerned as the extant literature did not provide clear-cut guidelines.

## 3. Materials and Methods

### 3.1. Participants

The participants of the study were 248 female freshmen who were enrolled in a written communication course devoted to research writing. The course was offered by a Saudi Arabian university conforming to a US curriculum and student-centered instruction. All participants were full-time students whose ages ranged from 18 to 25 years. The Office of the Registrar classified students as Arabic–English bilingual speakers. The choice of a convenience sample of female freshman students was based on practical considerations. First, due to the recent opening of academic programs to both male and female students, freshman female students were judged by faculty, administrators, and counselors as the most likely to need and benefit from interventions intended to offset failures and withdrawals. Second, the accessibility of male students to female researchers would have been challenging due to the largely gender-segregated campus.

### 3.2. Materials and Procedure

The course required students to carry out a study developed by the instructor and then write a paper on it in four parts (assignments 1–4), analyze the methodologies of published research reports (assignment 5), and complete a midterm and a final exam. Questions on both the midterm test and final test included a mixture of multiple-choice and short-answer questions. The latter presented subjects with simplified abstracts of published studies (i.e., research scenarios) in which students had to identify research questions, variables, hypotheses, designs, and/or results. The final test was a summative assessment tool to determine students’ comprehensive understanding of research methodologies in the social and behavioral sciences. The course was taught on campus by one instructor over the course of three semesters in sections of approximately 30 students each.

Before the final test, students were given practice sessions in class to dispel uncertainties regarding the format of the questions and the demands of the test. Tests administered in previous semesters were used for this purpose. Individual questions were displayed on a screen in the classroom where the course was offered. To simulate as closely as possible the experience of taking a test, after a question was presented to the class, each student was given 1 minute to answer in writing. Then, the instructor shared the correct answer, explained how specific information in the research scenario pointed to that answer, asked students to check their responses, and addressed any inquiries from the class. Students who attended all practice sessions and completed the course were included in the sample of 248 participants. The participation rate was 90.51%.

Following informed consent, students completed the short form of the Trait Emotional Intelligence Questionnaire (TEIQue–SF; Refs. [57,58]) and the Self-Efficacy Scale of Chen, Gully, and Eden [59]. The TEIQue–SF consisted of 30 statements (e.g., “I usually find it difficult to regulate my emotions”). Participants evaluated the extent to which each statement applied to them on a 5-point Likert scale ranging from “strongly disagree” (coded as −2) to “strongly agree” (coded as +2). On this scale, 0 served as the neutral point. The original 7-point scale was discarded in favor of a 5-point scale because prior work indicated that participants found the intermediate points unclear. The mean of each participant’s responses served as her global trait-emotional intelligence measure. Cronbach’s alpha, a measure of internal consistency, was 0.83.

The self-efficacy scale of Chen, Gully, and Eden et al. [59] was used to measure students’ overall confidence in their abilities to complete academic tasks. The scale entailed 8 generic statements of confidence (e.g., “When facing difficult tasks, I am certain that I will accomplish them”). Students were asked to focus on their academic abilities to determine the extent to which each statement applied to them. Students’ responses were made on a 5-point Likert scale from strongly disagree (coded as −2) to strongly agree (coded as +2). Thus, the general self-efficacy scale of Chen, Gully, and Eden [59] was adjusted to focus students’ attention on their abilities to complete academic tasks rather than on their abilities across undefined tasks. Cronbach’s alpha was estimated to be 0.87.

Students were asked to predict their final test grades both before and after taking the final exam. Students were given a sheet for making their grade predictions (see [60]) on a scale from 0 (minimum number of points to be obtained) to 15 (maximum number of points) as well as to express their subjective confidence in the predictions made on a 5-point Likert scale from 0 (not at all confident) to 4 (extremely confident). They were instructed to be realistic in their estimates rather than wishful. Students’ final test grades and class grades were then collected from the instructor at the end of the semester. Class grades were the composite outcome of 5 assignments, a midterm test, and a final test. The research was approved by the Deanship of Research, the ethical overseer at the selected institution. Debriefings were offered at the end of the semesters dedicated to data collection.

### 3.3. Data Analysis

After the records of each participant were linked, all identifying information was deleted. Codes produced by a random number generator were used to uniquely identify participants on data sheets. All estimates and grades were translated into percentages. Each student’s estimation bias was computed by subtracting the actual grades from the predictions made both before (prospective estimates) and after (retrospective estimates) the final test. A value with a + sign indicated an overestimation, a value with a − sign implied an underestimation, and a value equal to 0 reflected an accurate estimation. Instead, students’ reports of the extent to which such estimates were judged reliable (i.e., subjective confidence) were kept on the original 5-point scale (0–4). 

Following descriptive statistics, inferential statistics were intended to achieve the following aims: (a) to determine how the different measures were related to one another in the overall sample of participants; (b) to assess the extent to which differences existed between poor and satisfactory performers; and (c) to examine the extent to which academic performance (as measured by students’ final exam grades or class grades) could be predicted by particular individual differences. Included were estimation bias, subjective confidence in the estimations made, emotional intelligence (EI), and self-efficacy (SE) in one’s academic abilities broadly defined.

Both estimation bias and subjective confidence were treated as indices of students’ awareness of their preparation for the final test (i.e., a summative assessment measure). Students’ SE in their academic abilities was intended to offer a broader view of students’ confidence as learners, whereas EI was intended to provide some insights into students’ ability to gather information from their social environment (e.g., feedback from the practice sessions) to guide predictions and future behavior. All results were considered significant at *p* < 0.05.

## 4. Results

### 4.1. Description of Students’ Responses

Table 1 illustrates the mean (*M*) and standard deviation (*SD*) of academic performance measures (i.e., final test grades and class grades), and individual difference measures (i.e., bias in estimation and subjective confidence before the final test, bias in estimation and subjective confidence after the final test, EI, and SE).

A Pearson correlation coefficient assessed the relationship between the selected measures in the whole sample of participants (see Table 2). For each correlation coefficient, a coefficient of determination indicated the percentage of variance that any two measures shared, thereby illustrating the extent to which one measure could predict the other. 

Overestimation either before or after the final test was accompanied by a decline in final test grades [−0.72 and −0.61]. Instead, subjective confidence before or after the final test increased with students’ final test grades [+0.14 and +0.20]. Namely, poor-performing students might have overestimated their test performance, but they did not put as much confidence in their estimates as those with satisfactory grades. Although estimation biases and subjective confidence judgments concerned the final test, the same pattern applied to class grades. It was simply less pronounced. This pattern contradicted the claim that poor-performing students are unaware of their test preparation. Furthermore, there was no significant relationship between estimation biases and subjective confidence in the estimations made, suggesting that wishful thinking (as a motive driving students’ responses) applied to estimations, but not to confidence.

Both EI and SE were not significantly related to final test grades. However, as EI and SE increased, subjective confidence in the estimation made before the test increased too [+0.18 and +0.22]. The coefficients of determination, which indexed the percentage of variance in one measure that could be accounted for by another, were rather small, except for the relationship between the final test grades and students’ estimation biases.

### 4.2. Description of the Responses Given by Satisfactory and Poor Test Performers

Did the experience of completing the final test help all performers? To answer this question, students were divided into two performance groups by considering that at the selected university, 67% was defined as the lowest passing grade. Out of 248 students, 120 students qualified as satisfactory performers (≥67%), whereas 128 qualified as poor performers (<67%). A mixed factorial analysis of variance (ANOVA) with time (before and after) and performance group as the independent variables was carried out on both estimation bias and subjective confidence. Table 3 displays the descriptive statistics of the two performance groups.

Overall, students’ accuracy of estimation increased after having taken the final test [*F*(1, 246) = 106.44, *MSE* = 77.16, *p* < 0.001, *ηp^2^* = 0.302]. As expected, students with poor performance were less accurate than those with satisfactory performance [*F*(1, 246) = 129.74, *MSE* = 471.46, *p* < 0.001, *ηp^2^* = 0.345]. Although they overestimated their test grades, their estimations after the final test shrank twice as much as those of students with satisfactory performance [*F*(1, 246) = 10.53, *MSE* = 77.16, *p* < 0.001, *ηp^2^* = 0.041].

Across the entire sample, students’ subjective confidence decreased after the final test [*F*(1, 246) = 27.35, *MSE* = 0.603, *p* < 0.001, *ηp^2^* = 0.100]. Overall, poor performers were less confident than satisfactory performers [*F*(1, 246) = 5.24, *MSE* = 1.54, *p* = 0.023, *ηp^2^* = 0.021; other *F* < 1, *ns*].

In contrast to the lower SE of poor performers uncovered by Pilotti et al. [11], SE was not significantly different between performance groups [*F* < 1, *ns*; *M* = +0.42 and *SD* = 0.45; *M* = +0.47 and *SD* = 0.43]. EI exhibited the same pattern of non-significant differences [*F* < 1, *ns*; *M* = +0.84 and *SD* = 0.72; *M* = +0.86 and *SD* = 0.60].

Students’ pattern of responses involving estimation bias and subjective confidence did not fit the profile of students who were unaware of their test readiness. Both poor and satisfactory performers were able to improve their discrete estimates after having taken the test. They even moderated their subjective confidence in such estimates, further questioning the profile of poor performers as unaware of their test readiness. However, the lack of significant group differences in SE did not adhere to the profile of poor performers who are overall less confident in their academic abilities than satisfactory performers.

### 4.3. Do Individual Differences Contribute to Final Test Grades?

Lastly, a linear regression analysis was conducted with students’ estimation bias and subjective confidence before the final test, EI, and SE as the predictors, and final test grades as the outcome variable. The goal was to determine the unique contribution of each predictor when the contribution of the others was controlled. We chose to focus on students’ responses before the final test as they illustrated students’ expectations regarding their test readiness unadulterated by the experience of encountering the test. We conducted the same analysis with class grades as the outcome variable to identify the factors that would most effectively predict performance in a course. Table 4 illustrates the results of both analyses. In the table, the column before the last reports semi-partial correlations (i.e., the relationship between a predictor and the outcome variable while controlling for the other predictors). The corresponding coefficients of determination (i.e., the percentage of variance in the outcome variable that is accounted for by a given predictor while controlling for other predictors) are displayed in the last column.

No evidence of multi-collinearity (final test grades: tolerance values greater than 0.81; mean VIF = 1.13; class grades: tolerance values greater than 0.81; mean VIF = 1.13) was found. Estimation biases and subjective confidence predicted students’ performance on the final test in different ways. The magnitude of students’ overestimation predicted poor performance, whereas their subjective confidence predicted higher performance. Before the final test, EI also predicted higher performance. However, it is important to note that estimation bias provided the most substantive contribution to both test performance and class grades.

## 5. Discussion

The findings of the present study can be summarized in three main points: First, as the final test performance declined, the magnitude of the estimation bias before and after the test increased, whereas subjective confidence in the estimations made decreased. There was no relationship between estimation bias and subjective confidence, which suggests that students’ discrete outcome predictions might have relied on wishful thinking, whereas confidence judgments in such predictions might have embodied students’ attempts to be pragmatic.

Second, after participants had direct experience with the final test, they made more accurate grade estimations and became more conservative in the confidence they placed in their estimations. These findings indicate that students were able to process the information directly gathered from the test and used it to shape their self-evaluations of performance attainment. Students might have relied on wishful thinking more or less depending on their test readiness (as illustrated by their final test grades), but they were not insensitive to the reality check offered by the actual test. Thus, our finding conflicts with the profile of poor performers proposed by Jansen, Rafferty, and Griffiths [61], which implies insensitivity to the available evidence, and that suggested by Coutinho et al. [1], which implies overconfidence. 

Third, the best predictor of performance in the final test was the estimation bias before the final test, which accounted for 56% of the variance in participants’ performance. Students’ estimation bias before the test was again the best predictor of class performance, accounting for 16% of the variance. The other variables, such as subjective confidence, self-efficacy, or emotional intelligence, contributed either little or not at all to performance. These findings suggest that for interventions involving at-risk students, a brief quiz preceded by a grade-prediction exercise, administered early in the semester, might help faculty to forecast students’ performance in a course. Early detection is key but challenging when using traditional early course performance measures [62,63]. A brief exercise in grade prediction can offer valuable information to educators and counselors on the students who need additional support. 

The results of our study are consistent with earlier reports that poor performers tend to overestimate their likely attainment relative to higher performers but are less confident in their predictions [8,9,10,11]. In agreement with Pilotti et al. (2021), students are able to benefit from the experience of taking the final test, not only improving the accuracy of their estimations but also reducing their subjective confidence, a finding that makes the Dunning–Kruger effect [64] a somewhat malleable phenomenon. 

When extensive practice combines familiarization with test materials and ample response feedback, as in the present study, it is reasonable to ask whether practice impacts poor students’ desire to do well to the point of making them more attentive to their final test performance. If we compare the findings of Pilotti et al. [11], which do not contain this form of practice, with ours, the answer is affirmative. After having taken the final test, poor performers in the current study improved their estimates twice as much as those of Pilotti et al. (10.70% vs. 5.10%) [11] and reduced their subjective confidence much more (0.43 vs. 0.12). However, before the final test, practice with test questions and response feedback did not seem to help poor performers. Namely, with or without such practice, overestimations remained high and confidence low, thereby suggesting that the information gathered from practice may not be entered into forecasting until the final test is experienced. A reasonable explanation for this pattern of results can be found in students’ comments during debriefings. Before the final test was administered, students reported being doubtful that the final test would replicate much of the old tests used for practice. Thus, students may have resisted relying heavily on the information gathered from old tests for estimates and confidence judgments before the test.

Overall, our findings agree with the social–cognitive theory of Bandura [21], which posits the interaction of external and internal sources to account for students’ motivation and regulation of their conduct [22,23,24]. Indeed, in our study, an undesirable proclivity, often associated with poor performance (i.e., estimation bias), is found to be sensitive to information gathered from experience (i.e., the final test and, to a certain degree, practice with feedback). Our findings also illustrate human agency at work (including the properties of intentionality, forethought, and self-reactiveness; [65]). Students use information independently, choosing to rely on some information (practice with old tests) for forecasting only after they become convinced that it is valuable for that particular purpose (i.e., after having encountered the final test). 

Our study has limitations that will likely foster further research. First, the generalizability of its findings is to be assessed in courses other than the selected one. That is, courses that may be different in content, level of difficulty, and instructional format (e.g., online versus face-to-face). Second, the main reason for our selecting female freshmen, besides their availability, was that they were identified by faculty, administrators, and counselors as the most likely to benefit from interventions intended to offset failures and withdrawals. Our decision was also driven by the recognition of the crucial role that young female college students are expected to play in the national economic plan called Vision 2030. They are considered the main pillars of the plan that is intended to move Saudi Arabia from an oil-based economy to one that is knowledge- and service-based [66]. Notwithstanding the contextual relevance of young women, the current study’s sample of exclusively female freshman students may question whether its findings generalize to male students or other levels of educational attainment. Third, it may be of interest to identify dispositions other than emotional intelligence and self-efficacy that can modulate students’ performance predictions, such as humility [67]. The null or weak relationships between students’ estimates or confidence in such estimates and the selected dispositions (i.e., emotional intelligence and self-efficacy) also need to be further investigated to determine what particular instructional conditions (e.g., practice with abundant feedback) make such dimensions less useful. Fourth, the extent to which wishful thinking during forecasting satisfies students’ self-serving biases is to be determined. Self-serving biases are embodied in the propensity to associate oneself with desirable events and outcomes and detach oneself from undesirable events and outcomes [68]. Although further research is needed, it will rest on the assumption that a lack of awareness is unlikely to be the main motive behind poor performers’ difficulties in forecasting test outcomes.

## Figures and Tables

**Table 1:** Descriptive statistics.

*Measures*	*M*	*SD*
Bias in Est. Before the FT (0–100)	+21.28	20.25
Subjective Conf. Before the FT (0–4)	2.44	0.98
FT grade (0–100)	64.21	20.13
Bias in Est. After the FT (0–100)	+13.06	19.68
Subjective Conf. After the FT (0–4)	2.08	1.10
Class grade (0–100)	84.52	10.84
EI (−2–+ 2)	+0.45	0.44
SE (−2–+ 2)	+0.85	0.66

Note: FT = Final Test.

**Table 2:** Pearson correlation coefficients.

	EB	CB	FT	EA	CA	CG	EI	SE
EB		*ns*	−0.72 51.84%	+0.80 64.00%	*ns*	−0.38 14.44%	*ns*	*ns*
CB			+0.14 1.96%	*ns*	+0.45 20.25%	+0.13 1.69%	+0.18 3.24%	+0.22 4.84%
FT				−0.61 37.21%	+0.20 4.00%	+0.63 39.69%	*ns*	*ns*
EA					*ns*	−0.33 10.89%	*ns*	*ns*
CA						+0.16 2.56%	*ns*	*ns*
CG							*ns*	*ns*
EI								+0.41 16.81%

Note: Estimation Bias Before the FT =EB; Subjective Confidence Before the FT = CB; FT = Final test; Estimation Bias After the FT = EA; Subjective Confidence After the FT = CA; Class Grade = CG; EI = Emotional Intelligence; SE = Self-Efficacy. Non-significant correlation = *ns*.

**Table 3:** Mean and standard deviation of estimation accuracy and subjective confidence as a function of performance group.

	Poor Perform.		Satisfactory Perform.	
	*M*	*SD*	*M*	*SD*
Bias in Est. Before the FT	33.27%	19.38	8.49%	11.41
Bias in Est. After the FT	22.57%	19.96	2.91%	13.34
Change	10.70%		5.58%	
Subjective Conf. Before the FT	2.35	1.01	2.54	0.94
Subjective Conf. After the FT	1.92	1.06	2.24	1.12
Change	0.43		0.30	

**Table 4:** Regression analysis with final test grades or class grades as the outcome variable.

Final Test Grades	B	Std. Error	Beta	*t*	Sign.	Semi-Part. Corr.	%
Constant	67.91	2.43					
Bias in Est. Before FT	−0.75	0.04	−0.75	−18.01	<0.001	−0.75	56%
Subjective Conf. Before FT	4.48	0.88	0.22	5.08	<0.001	+0.21	4%
EI	5.05	2.10	0.11	2.41	= 0.02	+0.10	1%
SE	−1.19	1.40	−0.04	−0.85	*ns*		
**Class Grades**							
Constant	83.90	1.83					
Bias in Est. Before FT	−0.22	0.03	−0.40	−6.85	<0.001	−0.40	16%
Subjective Conf. Before FT	1.88	0.67	0.17	2.82	= 0.005	+0.16	3%
EI	1.79	1.59	0.07	1.13	*ns*		
SE	−0.24	1.06	−0.02	−0.23	*ns*		

Note: Before the test: *R* = 0.76; after the test: *R* = 0.43.

## Data Availability

Data are available upon request.
